# Legal Pharmacy

**Published:** 1907-10

**Authors:** 


### Legal Pharmacy

Meyers Brothers' *Druggist* has started a department of legal pharmacy. This department is under the efficient editorship of Mr. L. Frank Ottofy, of St. Louis. Among the interesting items contained in the July number is the citation of a case in New York in which a woman applied to a pharmacist for something with which to wash out a cut on her husband's knee. He gave her a bottle on which, among others, was the words "Poison, carbolic acid." The knee after the use of this turned black and caused serious trouble. It was found on analysis to contain between 86 and 90% of carbolic acid. The druggist was sued for damages and a verdict was given in the lower court for the plaintiff, the court holding that it was negligence for the clerk to sell a preparation of such dangerous character as was furnished in this case on a request from a customer. The court held that the remedy, if not efficient, should at least be harmless. It was held that the act of the clerk was chargeable to the master, and this decision, holding the druggist liable in damages, was confirmed by the Supreme Court of New York.

